# Survival benefit of gastrectomy for gastric cancer with peritoneal carcinomatosis: a propensity score‐matched analysis

**DOI:** 10.1002/cam4.877

**Published:** 2016-09-20

**Authors:** Xiuwen Geng, Hao Liu, Tian Lin, Yanfeng Hu, Hao Chen, Liying Zhao, Tingyu Mou, Xiaolong Qi, Jiang Yu, Guoxin Li

**Affiliations:** ^1^ Department of General SurgeryNanfang HospitalSouthern Medical UniversityGuangzhouChina; ^2^ Department of Gastrointestinal SurgeryThe First People's Hospital of YueyangYueyangChina

**Keywords:** Chemotherapy, gastrectomy, gastric cancer, peritoneal carcinomatosis

## Abstract

Peritoneal carcinomatosis (PC) is the most frequent pattern of metastasis in stage IV gastric cancer (GC). The study aims to investigate the efficacy of gastrectomy in GC with PC. Clinicopathological data of 518 stage IV GC patients were retrospectively collected in Nanfang Hospital. Among all cases, 312 GC patients with PC (without other site of metastasis) were eligible. Univariate and multivariate analyses were performed to identify the independent prognostic factors. Propensity score matching analysis was performed to balance the characteristics and treatment‐related factors. There was a significantly improved overall survival in gastrectomy group (148 patients) compared with nonresection group (164 patients) (*P *<* *0.001). The 1‐year and 2‐year survival rates were 49.8% and 21.5% in gastrectomy group, whereas 28.8% and 9.7% in nonresection group, respectively. Further analysis showed that gastrectomy had also improved survival in P1 (*P *=* *0.017) and P2 stage patients (*P *<* *0.001), but not P3 stage (*P *=* *0.495). The modality of gastrectomy plus chemotherapy plus hyperthermic intraperitoneal chemotherapy (HIPEC) showed an optimum survival. In addition, P3 disease, nongastrectomy, nonchemotherapy, non‐HIPEC, and age ≥ 60 years were independently associated with poor survival. The gastrectomy plus chemotherapy plus HIPEC modality showed a significant survival benefit for gastric adenocarcinoma patients, particularly in those with P1 and P2 diseases.

## Introduction

Gastric cancer contributes significantly to the burden of cancer‐related death worldwide and in China, mainly because of most patients being presented with advanced disease at the time of diagnosis 1, 2. According to the most recent statistical data of National Central Cancer Registry of China, both the incidence rate and mortality rate of gastric cancer are estimated to rank the second highest among cancers in 2015 3. Besides, about 679,100 Chinese would be newly diagnosed gastric cancer and about 498,000 Chinese would die from gastric cancer in 2015. Highly advanced gastric cancer characterized by local invasion and/or metastasis has dismal prognosis, which, is particularly unfavorable for patients with peritoneal carcinomatosis (PC) and almost 60% of gastric cancer deaths are due to PC 4. As the most frequent pattern of metastasis 5, 6, PC was observed in 10–20% of patients who were scheduled for potentially curative resection and 40% of patients who were clinically staged as II–III before the intraoperative abdominal examination 7. In the literature, the median survival time (MST) of gastric cancer patients with PC is 3–9 months 8, 9.

In recent years, the overall survival rate of gastric cancer patients increased with the availability of new medicine and combined chemotherapy compared with previous supportive treatment 8. Despite the improvement of systemic chemotherapy, the long‐term survival rate of patients with PC is still very low 9, 10. Additionally, the acquired resistance and adverse effects limit chemotherapy. Surgical resection has been considered as the most efficient treatment for early gastric cancer patients for a long time. For patients with advanced disease, it is generally agreed that surgery provides palliation of the major symptoms such as bleeding and/or obstruction, although the optimal surgical management for patients with minimal symptoms is debated. The National Comprehensive Cancer Network recommends that patients with metastatic disease are not candidates for surgery unless they present with bleeding and/or obstruction. However, in 2011, the Japanese Gastric Cancer Association guidelines suggested that patients with metastases might be candidates for gastrectomy even without major symptoms 11, 12.

As PC is currently regarded as a variant of systemic spread disease of gastric cancer, systemic chemotherapy is considered as the main treatment modality 11, 12. It is controversial if gastrectomy is beneficial for gastric cancer patients with PC. In the past few years, a few groups of surgeons investigated the feasibility of noncurative gastrectomy in patients with incurable factors 13, 14. Studies suggested that gastrectomy could raise the survival rate of the patients with PC without increasing the mortality rate and might be beneficial to reduce symptoms and enhance life quality 15, 16, 17. Other reports, however, had indicated that palliative gastrectomy was associated with significant morbidity, longer hospital stays, poor quality of life, and had no survival benefit in these patients 18, 19. In 2014, the GYMSSA (NCT00941655) trial reported that maximal cytoreductive surgery combined with hyperthermic intraperitoneal chemotherapy (HIPEC) and systemic chemotherapy could achieve more prolonged survival in selected gastric cancer patients with PC than the chemotherapy group 20. However, the recent results of REGATTA (UMIN000001012) trial presented that gastrectomy followed by chemotherapy did not show any survival benefit compared with chemotherapy alone in the advanced gastric cancer patients with a single noncurable factor 21. Hence, there is still no consensus on the value of gastrectomy for late‐stage gastric cancer patients with PC.

This study aimed to evaluate the impact of gastrectomy on the survival of stage IV gastric cancer patients with PC from the results of a retrospective cohort in a single medical center in China.

## Patients and Methods

### Cohort

A total of 4135 patients were diagnosed with gastric cancer in Nanfang Hospital, Southern Medical University, Guangzhou, China, from January 2005 to September 2015. The study was approved by Institutional Review Board of Nanfang Hospital, Southern Medical University. Among these patients, 1115 (27.0%) were classified as stage IV adenocarcinoma according to the third English edition of the Japanese classification of gastric carcinoma 11. After reviewing the medical records of these patients retrospectively by the two independent surgical oncologists, there were five categories of patients unqualified for this study: 108 patients (9.7%) with incomplete medical records data (including patients without the detailed description of the peritoneal metastasis in the operative reports); 214 patients (19.2%) without follow‐up data; 198 patients (17.8%) who refused to accept further advanced therapy when the disease was diagnosed; 15 patients (1.3%) who passed away during the first course of hospitalization; and 62 patients (5.6%) who were diagnosed and treated in other hospitals previously.

**Figure 1:**
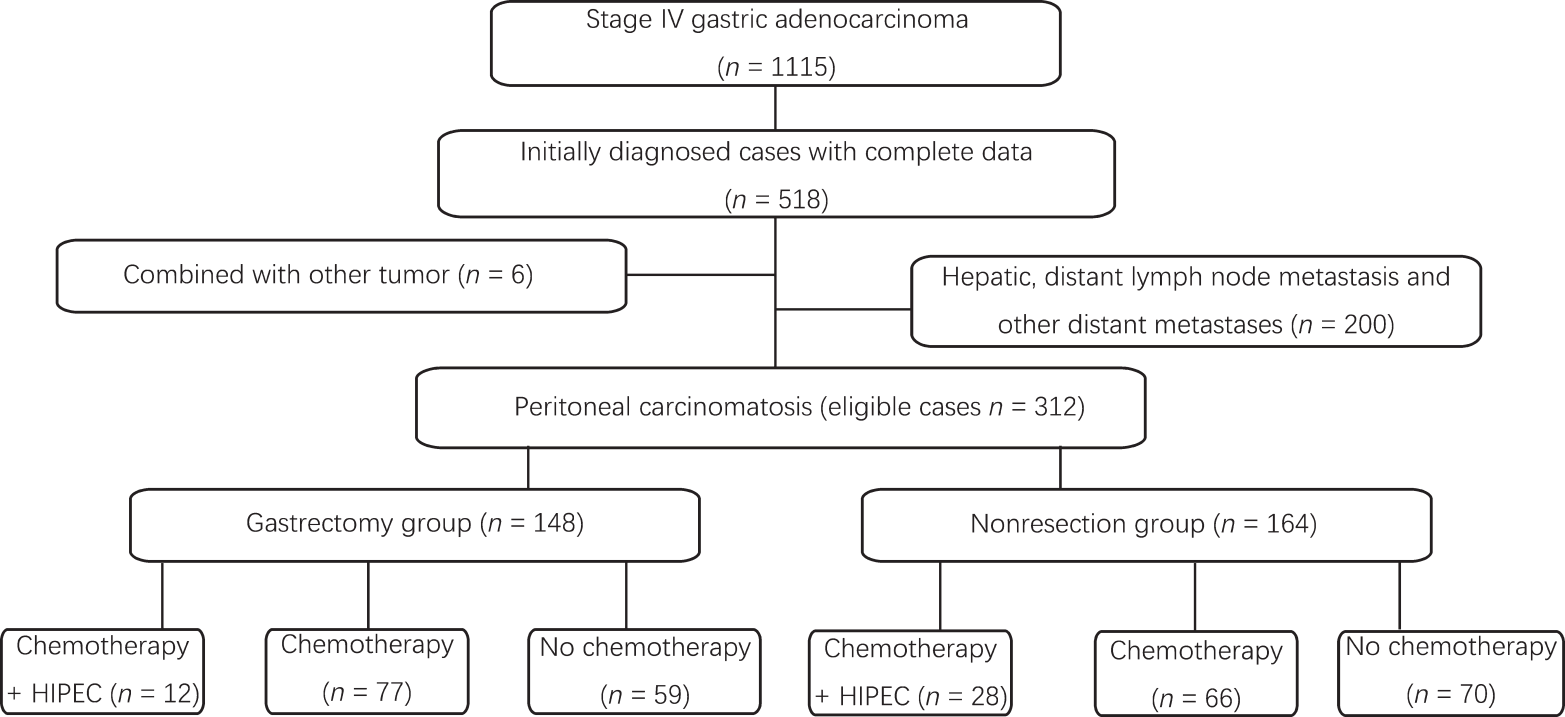
Selection and grouping of stage IV gastric adenocarcinoma patients with peritoneal carcinomatosis.

After the above exclusion, 518 patients were enrolled. As our study was focused on the gastric cancer patients with PC only, 6 patients who combined with other tumors (0.5%) and 200 patients with hepatic/distant lymph node metastasis or other distant metastases (17.9%) were further excluded. At last, 312 patients were eligible for this study. These enrolled patients were divided into a gastrectomy group and a nonresection group based on whether the gastrectomy was performed or not. Patients who underwent gastrojejunostomy or only exploratory laparotomy were classified into the nonresection group. Each group was further divided into three subgroups according to the treatment strategies: chemotherapy plus HIPEC, chemotherapy, and no chemotherapy (Fig. 1). Then, the 312 patients were divided into three subgroups in accordance with the extent of PC. In these three groups, each group was further divided into a gastrectomy subgroup and a nonresection subgroup. The patients' clinicopathologic features, treatment‐related factors, and survival curves were compared between these groups and subgroups.

### The diagnosis of PC and definition of P stages

All patients were histologically diagnosed as gastric adenocarcinoma after endoscopy and biopsy before initial treatment and diagnosed histopathologically after laparotomy at the department of pathology, Nanfang Hospital, Southern Medical University.

On the basis of the operative findings, PC was classified according to the first English edition of Japanese classification of gastric carcinoma as follows: P0, no peritoneal seeding; P1, disseminating metastasis to the region directly adjacent the peritoneum of stomach (above the transversecolon), including the greater omentum; P2, several scattered metastases to the distant peritoneum and ovarian metastasis alone; and P3, numerous metastases to the distant peritoneum 22.

### Gastrectomy, chemotherapy, and HIPEC

Noncurative gastrectomy for gastric adenocarcinoma is defined as a gastrectomy either with a residual tumor of metastatic lesion or with an unresectable lesion 23. In this study, total or distal gastrectomy was performed according to the location of the primary lesion. Consequently, primary tumors and greater omentum were all removed regardless of lymphadenectomy or metastasectomy. Gastrectomy was performed in the following two conditions: first, feasibility was evaluated by operating surgeons based on patient's symptom, performance status, nutritional status, and technical feasibility; and second, the operation informed consent was signed preoperatively or intraoperatively. The circumstances below were considered to be contraindications to resection: infiltration of locoregionally advanced disease to the root of the mesentery, invasion or encasement of major vascular structures or important organs, and extensive adhesion or fixation of the tumor.

More than two cycles of chemotherapy were defined as chemotherapeutic intervention, and a 5‐fluorouracil‐based (93.3%) or paclitaxel‐based (6.7%) regimen was administered either preoperatively and/or postoperatively. HIPEC was performed by using a closed circuit of 120 mg docetaxel in 3500 mL normal saline at 43 ± 0.5°C for 60 min. The perfusion tubes were placed at appropriate sites during the primary operation and the HIPEC was conducted on day 2, day 4, and day 6 postoperatively.

### Follow‐up

The primary endpoint was MST. The secondary endpoints were the 1‐year overall survival (OS) rate and 2‐year OS rate. As of September 7, 2015, the average follow‐up duration was 11.9 months. Twelve patients in the gastrectomy group and six patients in the nonresection group were lost to follow‐up in the process of this study. Most complete data on prognoses were collected during the outpatient visit. Telephone calls and letters were used to identify patients who could not attend regular hospital visiting. In this study, OS time was defined as the period from initial treatment (surgery or chemotherapy) to the date of death. Patients who were still alive till the cutoff date, lost to follow‐up, and died of any other cause were marked as censored data.

### Propensity score matching analysis

We selected five covariates (age, Eastern Cooperative Oncology Group Performance Status [ECOG‐PS], P stage, chemotherapy, and HIPEC) for propensity score matching (PSM) analysis. The propensity score was calculated using a logistic regression model and a nearest neighbor matching algorithm. Patients from the gastrectomy group were one‐to‐one matched with patients from the unresection group based on the top 148 scores.

### Statistical analysis

Statistical analyses were performed with SPSS software version 21.0 for Windows (SPSS, Inc., Chicago, IL). The chi‐square test was used to compare the categorical variables and two independent *t*‐tests were used to compare the quantitative variables. Cumulative survival analysis was estimated using the Kaplan–Meier method and compared by the log‐rank test. Univariate and multivariate survival analyses were performed using Cox proportional hazards regression model to produce a hazard ratio. Hazard ratios and their 95% confidence intervals provided an evaluation of the relative rate of death between the two groups compared. Additionally, PSM analysis was conducted to balance the baseline characteristics and treatment‐related factors. Statistical significance was defined as *P *<* *0.05 (two sided).

## Results

### Baseline characteristics

A total of 312 patients were involved in this study and the comparable baseline clinicopathologic features and treatment‐related characteristic were all summarized in Table 1. Among these patients, the male and female ratio was approximately 1.8 (119/113) and their average age was 53.9 years old. More than 85% of patients were scored 0 and 1 of ECOG‐PS. The average Body Mass Index (BMI) was 20.8 kg/m^2^. The peritoneal dissemination was classified as P1 in 115 patients (36.9%), P2 in 69 patients (22.1%), and P3 in 128 patients (41.0%). The maximal Borrmann type of the tumor is type III (>70%), but type II is the least (<2%). All 312 cases are histopathologically adenocarcinoma and more than 90% are undifferentiated. Additionally, most patients (97.1%) did not receive targeted therapy, whereas 158 patients (50.6%) did not undergo palliative chemotherapy. Only 12.8% of patients experienced HIPEC. The overall postoperative morbidity rate is 11.2% (35/312) and postoperative complications including intraluminal hemorrhage, small bowel obstruction, wound problem, pulmonary infections, intraabdominal abscess, thromboembolism, etc., were observed. As shown in Table 1, the above characteristics were also compared between gastrectomy group (148 patients) and nonresection group (164 patients). There were no statistical differences between these two groups on patient's age, gender, BMI, Borrmannn type, tumor differentiation, the extent of peritoneal seeding (P stage), chemotherapy, targeted therapy, and postoperative morbidity. However, the differences between these two groups on ECOG‐PS and HIPEC were statistically significant (0.037 and 0.018, respectively).

**Table 1:** Clinical and pathologic characteristic of enrolled patients

Characteristic	Total (*n* = 312)		Gastrectomy*n* = 148 (51.3%)		Nonresection*n* = 164 (48.7%)		P value
	No.(%)	Mean ± SD	No.(%)	Mean ± SD	No.(%)	Mean ± SD	
Gender							0.539
Male	199 (63.8)		97 (65.5)		102 (62.2)		
Female	113 (36.2)		51 (34.5)		62 (37.8)		
Age, years		53.9 ± 13.5		55.3 ± 12.9		52.6 ± 13.8	0.081
ECOG‐PS							0.037
0, 1	274 (87.8)		136 (91.9)		138 (84.1)		
2, 3	38 (12.2)		12 (8.1)		26 (15.9)		
BMI, kg/m^2^		20.8 ± 3.0		20.9 ± 2.9		20.7 ± 3.0	0.503
Borrmann type							0.460
I	21 (6.7)		12 (8.1)		9 (5.5)		
II	3 (1.0)		1 (0.7)		2 (1.2)		
III	221 (70.8)		108 (73.0)		113 (68.9)		
IV	67 (21.5)		27 (18.2)		40 (24.4)		
Histology							0.489
Differentiated	22 (7.1)		12 (8.1)		10 (6.1)		
Undifferentiated	290 (92.9)		136 (91.9)		154 (93.9)		
Target chemotherapy							0.193
No	303 (97.1)		145 (98.0)		158 (96.3)		
Trastuzumab	2 (0.6)		0 (0.0)		2 (1.2)		
Cetuximab	2 (0.6)		2 (1.4)		0 (0.0)		
Bevacizumab	3 (1.0)		1 (0.7)		2 (1.2)		
Apatinib	2 (0.6)		0 (0.0)		2 (1.2)		
P Stage							0.185
P1	115 (36.9)		62 (41.9)		53 (32.3)		
P2	69 (22.1)		32 (21.6)		37 (22.6)		
P3	128 (41.0)		54 (36.5)		74 (45.1)		
Chemotherapy							0.115
Yes	154 (49.4)		80 (54.1)		74 (45.1)		
No	158 (50.6)		68 (45.9)		90 (54.9)		
HIPEC							0.018
Yes	40 (12.8)		12 (8.1)		28 (17.1)		
No	272 (87.2)		136 (91.9)		136 (82.9)		
Postoperative complication							
Intraluminal hemorrhage	4 (1.3)		3 (2.0)		1 (0.6)		0.266
Small bowel obstruction	7 (2.2)		5 (3.4)		2 (1.2)		0.199
Wound problem	3 (1.0)		1 (0.7)		2 (1.2)		0.623
Pulmonary infections	9 (2.9)		3 (2.0)		6 (3.7)		0.390
Intraabdominal abscess	5 (1.6)		5 (3.4)		0 (0.0)		0.018
Thromboembolism	6 (1.9)		4 (2.7)		2 (1.2)		0.194
Other	8 (2.6)		4 (2.7)		4 (2.4)		0.474
Total	35 (11.2)		22 (14.9)		13 (7.9)		0.052

HIPEC, hyperthermic intraperitoneal chemotherapy.

### Long‐term survival

**Figure 2:**
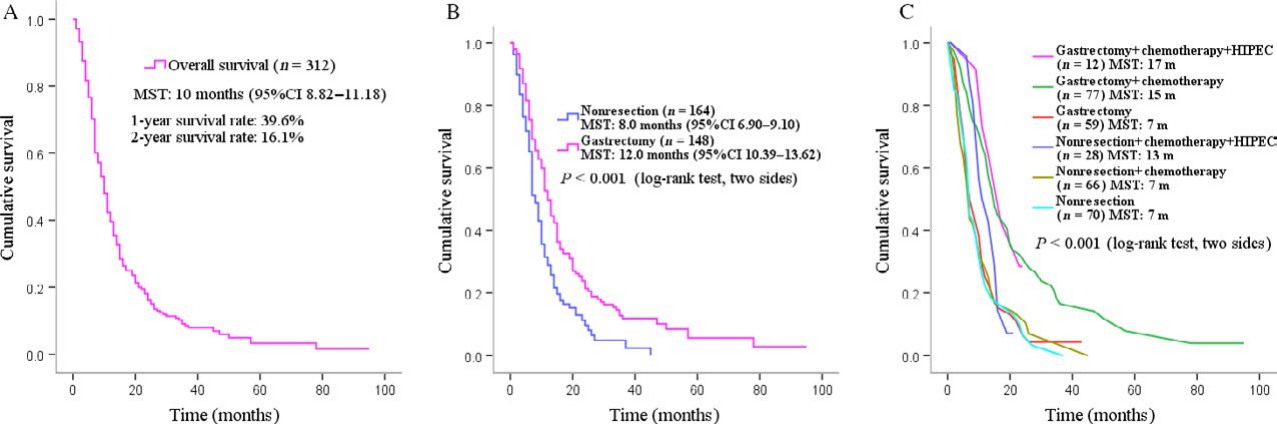
Overall survival and survivals of different treatment subgroups of stage IV gastric adenocarcinoma patients with peritoneal carcinomatosis.

Kaplan–Meier analysis showed the median survival for all 312 patients is 10.0 (95% confidence interval [95% CI]: 8.82–11.18) months. The 1‐year survival and 2‐year survival were 39.6% and 16.1%, respectively (Fig. 2A). After the patients were divided based on the surgery situations into gastrectomy group (*n* = 148) and nonresection group (*n* = 164), further analysis demonstrated that the median survival is 12.0 (95% CI: 10.39–13.62) months in the gastrectomy group and 8.0 (95% CI: 6.90–9.10) months in the nonresection group (*P *<* *0.001, Fig. 2B). The 1‐year survival rate is 49.8% in the gastrectomy group and 28.8% in nonresection group, whereas the 2‐year survival rate is 21.5% in gastrectomy group and 9.7% in nonresection group (*P *<* *0.001, Table 2).

**Table 2:** Comparison between the subgroups of treatment modality and P stages

Characteristic	Gastrectomy (*n* = 148)					Nonresection (*n* = 164)					*P* value
	*n*	MST (m)	95% CI	Survival rate (%)		*n*	MST (m)	95% CI	Survival rate (%)		
				1 year	2 year				1 year	2 year	
Treatment modality											
Chemotherapy + HIPEC	12	17.0	11.27–22.74	66.7	28.5	28	13.0	7.58–18.42	50.0	3.6	0.034
Chemotherapy	77	15.0	12.27–17.73	64.3	30.9	66	7.0	5.96–8.04	27.8	10.6	<0.001
No chemotherapy	59	7.0	4.66–9.34	24.9	6.6	70	7.0	6.32–7.68	22.2	6.6	0.691
P stage											
P1	62	16.0	12.29–19.71	69.0	31.5	53	12.0	8.49–15.51	47.0	25.7	0.017
P2	32	16.0	11.97–20.03	60.9	31.2	37	9.0	7.51–10.49	21.6	0.0	<0.001
P3	54	7.0	5.79–8.24	16.7	1.9	74	6.0	4.67–7.33	15.9	0.0	0.495
Overall	148	12.0	10.39–13.62	49.8	21.5	164	8.0	6.90–9.10	28.8	9.7	<0.001

MST, median survival time; HIPEC, hyperthermic intraperitoneal chemotherapy.

When the patients in gastrectomy group and nonresection group were further stratified into three subgroups by treatment modalities (chemotherapy plus HIPEC, chemotherapy, and no chemotherapy) (Table 2 and Fig. 2C), the results manifested that patients in gastrectomy group with either chemotherapy plus HIPEC or chemotherapy had statistically significant better survival rates than those in nonresection group, including MST (17.0 vs. 13.0 months, *P *=* *0.034; and 15.0 vs. 7.0 months, *P *<* *0.001), 1‐year survival rate (66.7% vs. 50.0% and 64.3% vs. 27.8%), and 2‐year survival rate (28.5% vs. 3.6% and 30.9% vs. 10.6%). However, no differences were observed in the subgroups of patients without chemotherapy between the gastrectomy group and the nonresection group (MST: 7.0 vs. 7.0 months, *P *=* *0.691).

When all the six subgroups were compared with each other (Table 2 and Fig. 2C), the results indicated that the treatment modality of gastrectomy plus chemotherapy plus HIPEC (*n* = 12, MST: 17.0 months) had the optimum survival when compared with the gastrectomy plus chemotherapy (*n* = 77, MST: 15.0 months) and gastrectomy alone (*n* = 59, MST: 7.0 months) in the gastrectomy group and all the three counterparts (*P *<* *0.001) in the nonresection group (nonresection plus chemotherapy plus HIPEC, *n* = 28, MST: 13.0 months; nonresection plus chemotherapy, *n* = 66, MST: 7.0 months; and no chemotherapy, *n* = 77, MST: 7.0 months). In addition, within the nonresection group, when the three subgroups were compared with each other, the patients with chemotherapy plus HIPEC showed a much significant survival benefit than the patients with only chemotherapy (MST: 13.0 vs. 7.0 months, *P *=* *0.037) and patients with no chemotherapy subgroups (MST: 13.0 vs. 7.0 months, *P *=* *0.044) (Table 2).

**Figure 3:**
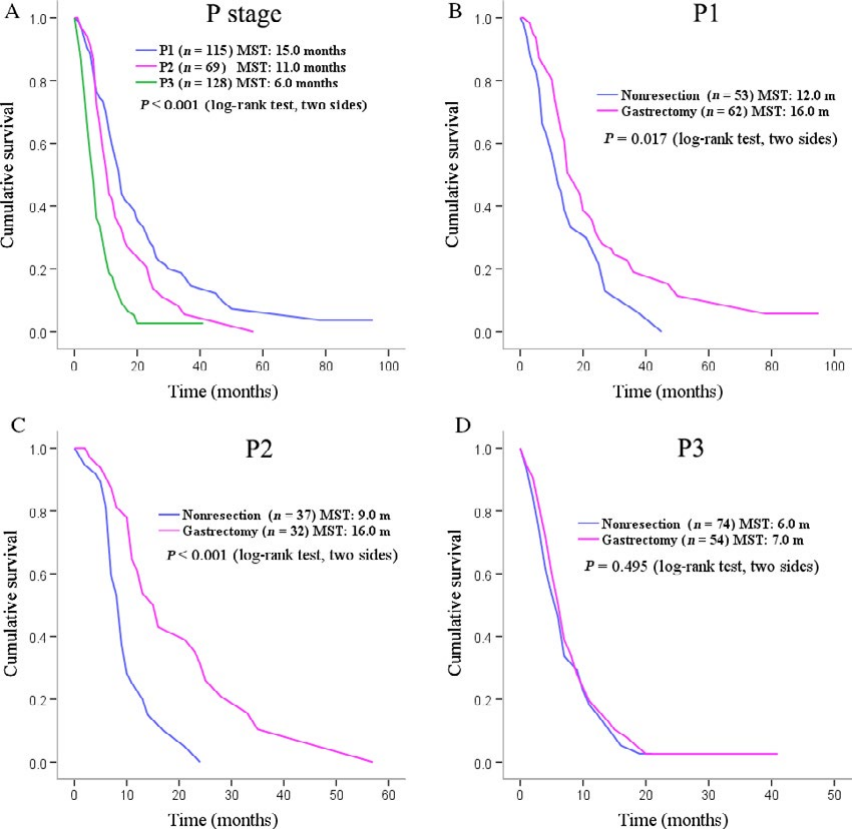
Overall survival and P stage subgroup survivals of stage IV gastric adenocarcinoma patients with peritoneal carcinomatosis.

When the OS was analyzed according to the extent of PC, it was unsurprising to find that there were significant heterogeneity of survival time among patients in P1, P2. and P3 (MST: 15 months vs. 11 months vs. 6 months; *P *<* *0.001 by log‐rank test), as shown in Figure 3A and Table 2. For groups P1, P2, and P3, the 1‐year survival rates are 59.2%, 42.1%, and 17.6%, respectively (*P* < 0.001, respectively) and the 2‐year survival rates are 28.4%, 16.5%, and 0.8%, respectively (*P* < 0.001, respectively) (Table 2).

When patients in gastrectomy group and nonresection group were stratified by P stages and further analyzed, it was found that in P1 and P2 subgroups (Fig. 3B, C and Table 2), patients with gastrectomy had statistically significant longer survival time than those in the nonresection subgroups including MST (16.0 vs. 12.0 months, *P *=* *0.017; and 16.0 vs. 9.0 months, *P *<* *0.001), 1‐year survival rate (69.0% vs. 47.0% and 60.9% vs. 21.6%), and 2‐year survival rate (31.5% vs. 25.7% and 31.2% vs. 0.0%). These differences were not observed between the gastrectomy subgroup and the nonresection subgroup of P3 patients, as shown in Figure 3D and detailed in Table 2.

### Propensity score matching analysis

To obtain more reliable evidence, PSM was conducted to compensate for selection bias and avoid potential confounding effects. The *P* values of ECOG‐PS and HIPEC between the gastrectomy group and nonresection group in the initial baseline were 0.037 and 0.018. After PSM38, the baseline and treatment‐related characteristics were balanced and patients were one‐to‐one matched between the two groups. The *P* values were recalculated and there were no significant differences between the gastrectomy group and nonresection group (for ECOG‐PS: *P *=* *0.328; and for HIPEC: *P *=* *0.427). The survival comparison was also performed again between these two groups, and the survival benefit was still observed in the gastrectomy group (MST: 12.0 vs. 7.0 months, *P *<* *0.001).

### Multivariate analysis

To explore an optimization model for which patients could benefit from the treatment of gastric adenocarcinoma with PC, analyses of univariate, multivariate, and prognostic factors were conducted in this study. Univariate analysis of potential prognostic factors was conducted for the 312 patients. In the analysis, patient's gender, age (<60 vs. > = 60 years old), ECOG‐PS (0, 1 vs. 2, 3), P stage (P1, P2, vs. P3), gastrectomy (yes or no), chemotherapy (yes or no), HIPEC (yes or no), and targeted therapy (yes or no) were enrolled as covariates. The univariate survival analysis revealed that, as shown in Table 3, age, P stage, gastrectomy, chemotherapy, and HIPEC were associated with survival. The following factors were considered to be irrelevant: gender (*P *=* *0.850), ECOG‐PS (*P *=* *0.906), and targeted therapy (*P *=* *0.666). In multivariate analysis, the patient's age ≥60 years old (HR: 1.561; 95% CI: 1.201–2.028; *P *=* *0.001), P3 disease (HR: 2.698; 95% CI: 2.046–3.559; *P *<* *0.001), nonresection (HR: 0.597; 95% CI: 0.456–0.781; *P *<* *0.001), nonchemotherapy (HR: 0.624; 95% CI: 0.479–0.814; *P *<* *0.001), and non‐HIPEC (HR: 0.539; 95% CI: 0.344–0.843; *P *=* *0.007) were identified as independent poor survival factors.

**Table 3:** Independent prognostic factors on the univariate and multivariate analysis

Characteristic	*n*	MST (m)	Univariate analysis			Multivariate analysis		
			HR	95% CI	*P* value	HR	95% CI	*P* value
Gender, male/female	199/113	10.0/10.0	0.975	0.747–1.271	0.850			
Age, <60/> = 60 years	196/116	11.0/8.0	1.450	1.121–1.874	0.005	1.561	1.201–2.028	0.001
ECOG‐PS, 0–1/2–3	274/38	10.0/10.0	1.023	0.701–1.492	0.906			
P Stage*,* P1, P2/P3	184/128	13.0/6.0	2.816	2.148–3.691	<0.001	2.698	2.046–3.559	<0.001
Gastrectomy, Yes/No	148/164	12.0/8.0	0.596	0.460–0.773	<0.001	0.597	0.456–0.781	<0.001
Chemotherapy*,* Yes/No	154/158	13.0/8.0	0.544	0.420–0.705	<0.001	0.624	0.479–0.814	<0.001
HIPEC, Yes/No	40/272	14.0/9.0	0.638	0.411–0.992	0.046	0.539	0.344–0.843	0.007
Target chemotherapy, Yes/No	9/303	15.0/10.0	0.847	0.399–1.799	0.666			

MST, median survival time, HIPEC, hyperthermic intraperitoneal chemotherapy.

## Discussion

This study showed that median survival was significantly longer in the gastrectomy group compared with the nonresection group in gastric cancer patients with PC. The 1‐year and 2‐year survival rates were also significantly higher in gastrectomy group compared with the nonresection group (28.8% and 9.7%, respectively). These results were supported by a report published by Xia et al., who found that the OS of patients with gastrectomy was longer than patients with nonresection group 15. Our findings were also consistent with the reported data of Sun et al., Hioki et al., Kim et al., and Li et al. 16, 24, 25, 26. In further analysis of the subgroups stratified by the extent of PC, we found that patients with P1 and P2 diseases had better median survivals than patients with P3 disease. Besides, compared with nonresection, gastrectomy was beneficial for patients with P1 and P2 diseases rather than P3 disease. These results were also similar to the previous reports 15, 27, 28.

Previous studies reported that gastric resection not only could prolong the survival of gastric cancer patients with PC but also reduced symptoms and improved life quality without increasing the mortality rate 17, 23. Theoretically, it is believed that cancer patients can benefit from the removal of tumor mass through multiple ways. First, it can reduce the local complications caused by the primary disease; second, it can reduce the resource of additional metastasis; third, when the tumor load is removed, the residual cancer cells might be more sensitive to the chemotherapeutics, making the response to chemotherapy increased; fourth, gastrectomy can relieve the metabolic demands from the tumor; and finally, patients may profit immunologically from decreased tumor burden as the cancer cells can produce some immunosuppressive cytokines 25, 29. In spite of these theoretical advantages and a few reports on the patients' survival benefits, gastrectomy for gastric adenocarcinoma patients with PC still remains a controversy and there are debates on the treatment modality in the literature. The REGATTA trial by Fujitani et al. 21 investigated the role of gastrectomy in management of advanced gastric cancer with a single incurable factor. The results showed that gastrectomy followed by chemotherapy did not display any survival benefit. They reported a surprisingly high median OS (16.6 months) for patients assigned to chemotherapy alone group, which was much higher than all the previous reported eight‐phase III trials performed from 2005 to 2014 for stage IV gastric cancer treatment 30. Moreover, they reported a 14.3‐month median OS for those assigned to gastrectomy plus chemotherapy. The REGATTA trial was a prospective study on stage IV gastric cancer with different metastatic sites, using a different chemotherapy regimen. There were no results of subgroup analysis for PC in the REGATTA trial. We remain in doubt if the different conclusions between REGATTA trial and all the other studies were caused by the different chemotherapy regimens or way of patients' selection. For example, in our study the gastric cancer patients were with PC and no other sites of metastasis.

Gastric cancer patients with PC only are a specific population. The tumor cells either directly perforate the entire gastric wall or through other ways to spread to the peritoneum before other distant metastasis. In our study, we selected 312 cases with complete clinical data from 518 initially diagnosed stage IV gastric adenocarcinoma patients. In these cases, the tumors were mostly located in the middle and lower parts of the stomach (about 88%) and more than 90% were Borrmann types III and IV. In approximately 95% of our cases, the adenocarcinoma was undifferentiated. Because of relatively poor blood circulation and low drug concentration in the peritoneum, tumor cells spreading to the peritoneum have less response to the general systemic chemotherapy than other organs, which makes it an obstacle of treatment. Recently, the implementation of a multimodality treatment including noncurative gastretomy combined with HIPEC has led to promising results in selected gastric adenocarcinoma patients with PC 20, 31, 32, 33, 34. A systematic review and meta‐analysis of 13 randomized controlled trials with acceptable quality have been established. It was concluded that HIPEC was associated with marked improvement in survival of advanced gastric cancer, in comparison with other current standard treatments 35.

In this study, we also analyzed the effects of different treatment modalities on patient's survival. We found that patients in gastrectomy group with either chemotherapy plus HIPEC or chemotherapy had statistically significant better survival rates than those in the nonresection group (MST: 17.0 vs. 13.0 months, and 15.0 vs. 7.0 months). However, these differences were not observed in patients without chemotherapy in gastrectomy group and nonresection group (MST: 7.0 vs. 7.0 months). These results indicated that patients did not benefit from gastrectomy without chemotherapy. We also compared the survival rates of patients who accepted the six different treatment strategies (Table 2). The results showed that the treatment modality of gastrectomy plus chemotherapy plus HIPEC (MST: 17.0 months) had the optimum survival when compared with gastrectomy plus chemotherapy (MST: 15.0 months) and gastrectomy alone (MST: 7.0 months) in the gastrectomy group and the three counterparts in the nonresection group. These results were similar to the report of Rudloff et al. in the GYMSSA trial 20, in which cytoreductive surgery combined with intraperitoneal and systemic chemotherapy in selected patients with PC achieved a prolonged survival. Another prospective randomized study conducted by Yang et al. 31 reported that patients in the gastrectomy plus HIPEC group had a longer median survival than those in the gastrectomy alone group (11.0 vs. 6.5 months) and several cohort studies also demonstrated that gastrectomy plus HIPEC could improve outcome of gastric cancer patients with PC 32, 33, 36. The combination of treatment modalities (gastrectomy plus chemotherapy plus HIPEC) had showed advantages and was currently favored by more oncological surgeon while a panel of international experts strongly recommended cytoreductive surgery plus HIPEC to be the current standard treatment for advanced gastric cancer (GC) 37, 38.

In our study, interestingly, when we analyzed the survivals in different subgroups within the nonresection group patients, we found that the chemotherapy plus HIPEC subgroup had a more significant survival benefit than the chemotherapy subgroup (MST: 13.0 vs. 7.0 months) and no chemotherapy subgroup (MST: 13.0 vs. 7.0 months), which suggested that HIPEC was beneficial to the nonresection patients. In the gastrectomy group, we observed a significant prolonged survival when the chemotherapy plus HIPEC subgroup was compared with the no chemotherapy and no HIPEC subgroup (MST: 17.0 vs. 7.0 months). However, in the gastrectomy group, only a 2‐month difference in MST was observed when the chemotherapy plus HIPEC subgroup was compared with the chemotherapy subgroup (MST: 17.0 vs. 15.0 months). We believe that the effect of HIPEC observed in the nonresection group was due to the tumor mass in the peritoneal cavity. When the mass was removed, the survival benefit of HIPEC observed by Yang 31 was masked by the effects of systemic chemotherapy in the gastrectomy group in our study.

The limitations of our study are as follow: first, this study is a retrospective study and it is well known that possible detection bias and performance of analysis bias might exist. Our patients might have received a variety of treatments, including gastrojejunostomy or noncurative resection with or without preoperative and/or palliative chemotherapy, which may be relevance to the survival, although we did a very strict selection. The possibility of selection bias was also existed, that meant, less severe cases might have been selected for surgical resection, which would have resulted in a better survival outcome. To overcome these problems and obtain more conclusive results, a well‐designed, large‐scale randomized controlled trial is needed to explore the survival benefit of gastrectomy plus chemotherapy plus HIPEC compared with chemotherapy alone for gastric adenocarcinoma patients with PC.

For the classifications of PC, we used the first edition of the General Rules for Gastric Cancer Study that was considered too rough and not enough to quantify to a certain extent. The Peritoneal Cancer Index, described by Jacquet and Sugarbaker 7, 39, 40, is a system of staging PC and able to assess the carcinomatosis quantification and distribution. However, the peritoneal cancer index classification might be too complicated to be widely used. Therefore, a more practical classification for PC is expected. In China, up to now, when surgeons encounter a gastric cancer patient with PC, the decision of treatment strategy is still largely relied on the operator's experience. There is no standard protocol for the treatment of these patients. According to the solid data provided in the results of our study, the treatment strategy should be decided based on the extent of peritoneal seeding (P stage) and the findings during an exploratory laparotomy (suitable for resection or not).

In conclusion, the gastrectomy plus chemotherapy plus HIPEC modality showed a significant survival benefit for gastric adenocarcinoma patients, particularly patients with P1 and P2 diseases, but not for P3 patients. HIPEC was also beneficial for the nonresection patients. These results may shed light on the role of adjuvant surgery on the treatment of stage IV gastric cancer with PC.

## Conflicts of Interest

The authors have declared no conflicts of interest.
